# piRNAclusterDB 2.0: update and expansion of the piRNA cluster database

**DOI:** 10.1093/nar/gkab622

**Published:** 2021-07-24

**Authors:** David Rosenkranz, Hans Zischler, Daniel Gebert

**Affiliations:** Institute of Organismic and Molecular Evolution, Anthropology, Johannes Gutenberg University, Mainz 55099, Germany; Senckenberg Centre for Human Genetics, Facharztzentrum Frankfurt-Nordend gGmbH, Frankfurt am Main 60314, Germany; Institute of Organismic and Molecular Evolution, Anthropology, Johannes Gutenberg University, Mainz 55099, Germany; Institute of Organismic and Molecular Evolution, Anthropology, Johannes Gutenberg University, Mainz 55099, Germany; Department of Genetics, University of Cambridge, Downing Street, Cambridge CB2 3EH, UK

## Abstract

PIWI-interacting RNAs (piRNAs) and their partnering PIWI proteins defend the animal germline against transposable elements and play a crucial role in fertility. Numerous studies in the past have uncovered many additional functions of the piRNA pathway, including gene regulation, anti-viral defense, and somatic transposon repression. Further, comparative analyses across phylogenetic groups showed that the PIWI/piRNA system evolves rapidly and exhibits great evolutionary plasticity. However, the presence of so-called piRNA clusters as the major source of piRNAs is common to nearly all metazoan species. These genomic piRNA-producing loci are highly divergent across taxa and critically influence piRNA populations in different evolutionary lineages. We launched the initial version of the piRNA cluster database to facilitate research on regulation and evolution of piRNA-producing loci across tissues und species. In recent years the amount of small RNA sequencing data that was generated and the abundance of species that were studied has grown rapidly. To keep up with this recent progress, we have released a major update for the piRNA cluster database (https://www.smallrnagroup.uni-mainz.de/piRNAclusterDB), expanding it from 12 to a total of 51 species with hundreds of new datasets, and revised its overall structure to enable easy navigation through this large amount of data.

## INTRODUCTION

PIWI proteins and piRNAs represent a mainly metazoan system for the regulation of a range of target sequences (1–4), including transposable elements (5,6), protein-coding genes (7–11) and long non-coding RNAs (12). These targets can be regulated transcriptionally (13), as well as post-transcriptionally (6,14) and are recognized through sequence complementarity by piRNAs, which guide their associated PIWI proteins to their destination. While in vertebrates the PIWI/piRNA pathway is mostly restricted to the germline, in invertebrate groups such as arthropods and mollusks, piRNAs are in addition ubiquitously found in somatic tissues (15,16). Moreover, PIWI proteins and piRNAs were identified in somatic stem cells of sponges and cnidarians (17,18).

In general, piRNA-producing loci, called piRNA clusters, are considered to lie at the very center of the PIWI pathway (6). These loci are transcribed from one or from both DNA strands into large precursor RNAs, which in turn are processed into 23–31 nucleotide (nt) mature piRNAs as they are loaded onto PIWI proteins. Further, the ping pong amplification cycle, which acts during post-transcriptional silencing, additionally contributes to piRNA biogenesis (6,14). In a typical metazoan genome, up to a few hundred piRNA clusters can be identified, ranging in their size between a few thousand base pairs (kb) to more than 100 kb. Though these regions make up overall only small portions of a genome with 0.1–5%, they produce the vast majority of piRNAs. In *Drosophila*, over 90% of all sequenced germline piRNAs can be derived from these genomic loci (19). Similarly, in mammals, up to 95% of pachytene piRNAs are produced from clusters, while still >55% of pre-pachytene piRNAs can be attributed to these distinct loci (5). Generally, piRNA clusters are more or less dispersed in the genome, though they do not occupy similar regions in different phylogenetic groups. In flies, piRNA precursors stem from mostly pericentric heterochromatic loci (6), whereas in mammals, (pachytene) piRNA-producing loci are euchromatic A-MYB promoter-dependent RNA polymerase II transcription units (20).

In all species studied so far, piRNA clusters evolve rapidly, appearing and disappearing rather quickly on evolutionary time scales and evolving neutrally on the sequence level (21–23). Therefore, each species has a unique set of piRNA clusters with varying numbers of homologous clusters shared between lineages. Noteworthily, it has also been shown that piRNA clusters are highly divergent within species, such as observed in human (24). Additionally, in those clades in which somatic piRNAs are common, piRNA clusters show distinct expression levels in different tissues (19,16), similar to the differential activity at various developmental stages that is observed more broadly (5,13,25,26).

The first release of the piRNA cluster database (27), which was launched as a central resource for piRNA cluster research, comprised >100 Sequence Read Archive (SRA) datasets from 12 species. Noteworthily, while extensive analysis showed that many piRNA databases are contaminated with non-coding RNA (ncRNA) fragments, especially when concerning somatic tissues, the piRNA cluster database stands out with a remarkably low amount of such contaminations (28), due to our stringent criteria for piRNA definition and piRNA cluster identification (29). Further, while different databases for piRNA sequences are maintained (30–32), it is to date still the only database that is dedicated specifically to piRNA clusters. In recent years, the amount of small RNA sequencing data has drastically increased, while our understanding of the piRNA pathway and the expression of piRNAs has grown continuously. In order to incorporate this progress into the piRNA cluster database, we have now released a major update, which includes >350 SRA datasets from 51 species, comprising >15 000 piRNA clusters in total. This set of species contains mollusks, arthropods, fishes, amphibians, reptiles, birds and mammals. We further significantly improved our small RNA transcriptome analysis, which now includes an extensive set of non-coding RNAs, and we provide bibliographic information, as well as easy access to reference data used in our analysis.

## DATA COLLECTION AND ANALYSIS

### Dataset search and initial processing

**Figure 1.**
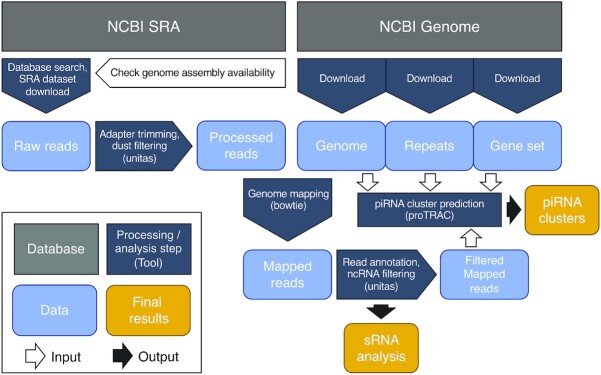
Processing and analysis pipeline for SRA dataset analysis and piRNA cluster prediction. The highlighted final results are accessible through the piRNA cluster database.

We systematically searched the NCBI sequence read archive (SRA) (33) for candidate small RNA sequencing datasets from a range of different tissue samples from metazoan species for which a reference genome is available. The raw sequence reads were first subjected to processing with unitas version 1.7.5 (34), including adapter trimming and low-complexity read filtering (Figure 1). The clean reads were then mapped with bowtie (35) to the corresponding reference genome, which was obtained from the NCBI Genome resource (36).

The mapped reads were annotated using unitas with reference datasets for coding sequences and non-coding RNAs, such as microRNAs, tRNAs, rRNAs and long non-coding RNAs. To determine the presence of piRNA-like reads, we examined the length distribution, rates of uridine at position 1 (1U) and adenine at position 10 (10A), as well as the rate of 10 nt 5′ read overlaps, known as ping-pong signature, of those reads that did not match to annotated RNAs in the previous step. The first threshold that was applied is a minimum share of piRNA-sized (23–31 nt) reads of 20%, while the largest peak was required to be within the piRNA size range. Additionally, since piRNAs typically show a high degree of sequence diversity with a considerable amount of unique sequence reads compared to other small RNA (sRNA) classes, a threshold for a minimum of 10% of unique reads within all piRNA-sized reads was employed. Finally, a 1U or 10A rate of at least 35% and a ping-pong signature with a significant (*P* < 0.05) *z*-score of >1.65 (37) were required to pass our filter.

### Genome mapping and piRNA cluster prediction

In preparation for the identification of piRNA clusters, we removed all previously annotated reads, as well as all reads that do not fall into the piRNA size range of 23–31 nt from the initial map file. A resulting map file with uniquely-mapping filtered reads without seed mismatches, produced by bowtie (35), and the corresponding genome was then used, along with an available repeatmasker annotation and a GFF gene set that were obtained from the NCBI Genome resource, to predict piRNA clusters with proTRAC version 2.4.4 (29). We applied a minimum cluster size of 5 kb and a minimum rate of 1U or 10A of 50%. Further, we used a sliding window size of 5 kb with an increment of 1 kb and defined a p-value of 0.01 for minimum number of normalized read counts per kb. To ensure similar cluster prediction power in all species, no assumptions on strand directionality were made (option: -clstrand 0). Finally, resulting piRNA cluster loci with a distance smaller than 10 kb were merged. However, if no piRNA clusters could be identified the dataset was discarded.

Overall, 358 SRA datasets from 51 species passed all filters and could be used for successful piRNA cluster prediction, totaling in 15 857 piRNA-producing loci with a median of 250 per species. The total number of unique clustered piRNA sequences amounts to nearly 88 million with a median of >1.1 million reads per species. All custom Perl scripts used in the data processing are available at GitHub (https://github.com/d-gebert/piRNAclusterDB).

## HOW TO ACCESS THE DATA

### Database structure and dataset access

**Figure 2.**
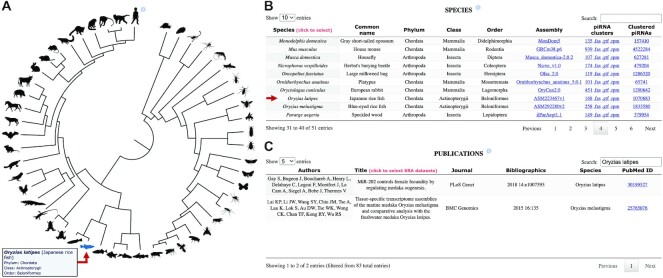
Top section and primary entry point of the piRNA cluster database. (**A**) Interactive phylogenetic tree, comprising the 51 species that are included in the database. (**B**) Complete tabular species list with quantitative information on piRNAs and piRNA clusters and associated download links, as well as links to the assemblies used in the analysis. (**C**) List of publications underlying the SRA datasets with PubMed IDs.

We completely redesigned the overall structure of the piRNA cluster database in order to accommodate to the vast number of datasets and species that we added in this major update. The primary entry point of the interface is the species selector, which is represented as an interactive phylogenetic tree (Figure 2A) on the one hand and as a tabular list on the other hand with additional information on taxonomy, number of piRNA clusters and total amount of clustered piRNA sequences (Figure 2B). The table also provides links to the corresponding genome assembly data that were used in our analysis, including genome, gene set (GFF) and repeatmasker file. A graphical representation of the locations of piRNA clusters on chromosomes is linked to the number of piRNA clusters in each species. Further, files on piRNA cluster coordinates (GTF), sequence (FASTA), and differential piRNA expression in reads per million (RPM) for each dataset, as well as pooled clustered piRNA reads are available for download. In addition to that, we provide a comprehensive list of all publications that are associated with small RNA (sRNA) datasets of the database, including PubMed IDs and direct links (Figure 2C).

### Cluster browser

**Figure 3.**
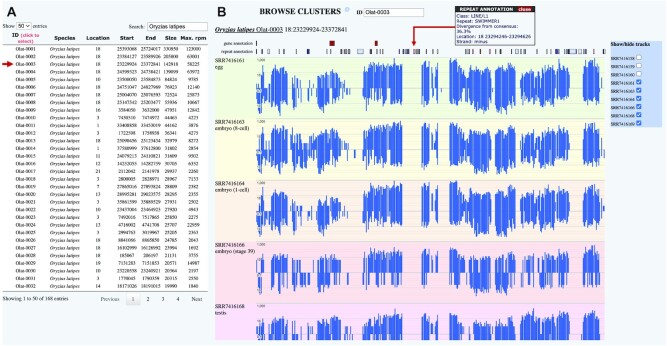
Cluster browser section of the piRNA cluster database. (**A**) List of all piRNA cluster loci of the selected species with coordinates, size and maximum rpm. (**B**) Browser with gene and repeat annotation tracks and piRNA clusters read coverage. Track elements are selectable for additional information.

Once a species of interest is selected, the user can browse piRNA-producing loci along all datasets from the chosen species in the cluster browser section (Figure 3). The available loci are provided in a list with selectable piRNA cluster IDs (Figure 3A), which contains additional information on location, size and reads per million. The cluster view incorporates tracks for gene and repeat annotation alongside of piRNA read coverage in rpm for plus and minus strands, which all can be individually inspected to receive further information, for example on gene or transposon name and repeat class, as well as exact rpm per position (Figure 3B). Finally, each SRA dataset can be individually selected and deselected to produce a customized view of piRNA cluster expression across different datasets, tissues, or developmental stages.

### SRA dataset section

**Figure 4.**
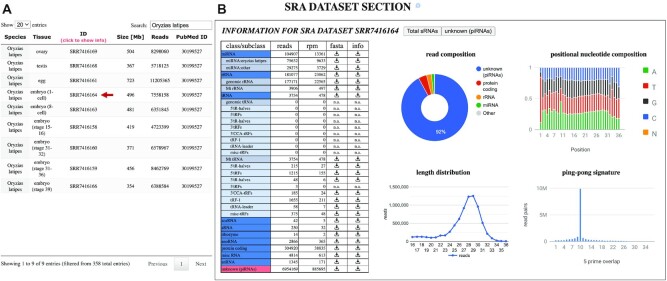
SRA dataset section of the piRNA cluster database. (**A**) List of SRA datasets of the selected species with information on tissue, data size, read count and PubMed ID. (**B**) Annotation of SRA datasets with download links for each RNA class and graphical output of analysis on read composition, positional nucleotide frequencies, length distribution and ping-pong signature (sense/antisense 5′ overlap lengths).

The third section of the database presents detailed analyses of processed, filtered, mapped and annotated sRNA reads for each SRA dataset from a selected species, which were generated by unitas (34). SRA datasets are selectable from a list that includes information on the tissue of origin, number of reads and PubMed ID of the associated publication (Figure 4A). For each of the annotated RNA types, such as miRNA, rRNA, tRNA-derived sRNA, lncRNA, mRNA and more, a table offers read counts, as well as links for the download of reads in FASTA format and info files with length distribution and positional nucleotide composition (Figure 4B). The fraction that most likely represents or contains to a large part piRNAs can be accessed under the type ‘unknown’, as these sequences could not be annotated as any other known sRNA. Moreover, graphical output on read composition, length distribution, positional nucleotide composition and ping-pong signature are provided for total sRNA reads and piRNA reads, which gives an accessible insight into the sRNA make-up of each SRA dataset and the contribution of piRNAs to the total pool of reads.

## DISCUSSION

Unsurprisingly, the first species in which piRNAs and piRNA clusters were identified and characterized were mice (*Mus musculus*) (1–4) and flies (*Drosophila melanogaster*) (6,14). Subsequent studies focused on other model organisms (38) and eventually extended towards non-model organisms, especially in recent years, including hitherto less well-studied taxa (15–18,25,26). Besides, many sRNA studies, e.g. in which miRNAs are the main focus, produced readily available piRNA fractions that were not yet examined.

This has created both the necessity and opportunity to considerably expand and restructure the piRNA cluster database to open up further potential for piRNA research. It is now clear that the PIWI pathway and piRNA-producing loci in particular evolve rapidly, leading to greatly different sets of clusters across taxa (21–23). The vast number of species included in this substantial update of the piRNA cluster database will enable evolutionary studies on an unprecedented scale, including previously less studied phylogenetic groups. In the past, studies in non-model organisms regularly yielded unexpected and novel insights into different aspects of the piRNA pathway (15,16,18,25).

Despite the considerable progress that has been achieved in recent years, many aspects especially concerning the evolution of piRNA clusters are still not fully understood. It has yet to be determined how exactly piRNA clusters initially emerge and what drives their genesis, their maintenance across evolutionary times and their demise. Furthermore, intriguing differences between phylogenetic groups, such as flies and mammals, regarding transposon enrichment or genomic location are likewise still not elucidated. For instance, it is conceivable that the very nature and origin of the transposon-rich, heterochromatic and pericentromeric piRNA clusters of *Drosophila* (6) are inherently different from those of mammalian clusters, which are more dispersed in the genome and less enriched for transposon sequences (5). Similarly, clusters of recently identified somatic piRNAs in non-vertebrates (15,16) remain to be thoroughly studied, to detect putative functions that might be different from those in gonads. Finally, differences in piRNA biology of male and female germline have not yet been widely studied but have so far yielded important insights (39). We believe that these outlined areas of research will greatly benefit from the resources that this update of the piRNA cluster database provides.
